# ﻿First mitogenome of the family Putoidae (Hemiptera, Coccomorpha) and its phylogenetic implications

**DOI:** 10.3897/zookeys.1247.144896

**Published:** 2025-07-22

**Authors:** Yu-ang Li, Xinyi Zheng, Han Xu, San-an Wu

**Affiliations:** 1 The Key Laboratory for Silviculture and Conservation of Ministry of Education, Beijing Forestry University, Beijing 100083, China Beijing Forestry University Beijing China

**Keywords:** Gene rearrangements, mitogenome, molecular phylogeny, Putoidae, scale insects, taxonomy

## Abstract

Scale insects are significant pests impacting agriculture, forestry, and ornamental plants. They play a dual role in ecosystems, serving as a food source for insects like bees, producing pigments and wax. Mitochondrial genomes have been widely utilized in phylogenetic studies. However, the mitogenomes of scale insects currently available in GenBank fail to adequately represent the majority of families. In this paper, the first complete mitogenome of *Putosinensis* Zheng & Wu, 2025 is described, revealing previously unreported gene rearrangements in scale insects. It has a length of 18,830 bp and a high A+T content of 90.7%. Moreover, the phylogenetic analysis based on mitogenomic sequences shows that archaeococcoids are a paraphyletic group, with the family Putoidae being sister to all neococcoids – a finding consistent with results from nuclear gene and morphological data. This underscores the utility of mitochondrial genome data in reconstructing phylogenetic relationships within the infraorder Coccomorpha.

## ﻿Introduction

Scale insects (Coccomorpha) belong to the suborder Sternorrhyncha within the Hemiptera, along with aphids (Aphidomorpha), whiteflies (Aleyrodomorpha), and psyllids (Psyllomorpha) (Gullan and Martin 2009). To date, Coccomorpha comprises approximately 8500 species in 56 families (36 extant + 20 extinct). Among these, approximately 640 species within 28 families are considered agricultural pests (García Morales et al. 2016; Kondo and Watson 2022). Taxonomically, Coccomorpha are divided into two informal groups: the archaeococcoids and the neococcoids. The archaeococcoids, typically feature abdominal spiracles in at least the females (Borchsenius 1958), and adult males possess either compound or a row of unicorneal eyes encircling the head (Williams et al. 2011). In contrast, the neococcoids lack the abdominal spiracles and compound eyes, typically having two pairs of simple eyes, except for Kermesidae and some Coccidae, which possess four or five pairs of simple eyes (Hodgson 2020).

Within Coccomorpha, Putoidae has faced contentious taxonomic placement since its establishment. Adult females of *Puto* exhibit morphological characteristics similar to those of adult females of *Phenacoccus* in Pseudococcidae. Therefore, Putoidae was often classified within Pseudococcidae and belongs to neococcoids (Borchsenius 1958; Tang 1992; Miller and Miller 1993; Gavrilov-Zimin and Danzig 2012). However, adult males of Putoidae possess an XX-X0 sex chromosome system (Hughes-Schrader 1948) and more than two pairs of unicorneal eyes (Koteja and Azar 2008; Powell and Miller 2024), whereas neococcoids typically exhibit a paternal genome elimination (PGE) system (Nur 1980) and only two pairs of simple eyes (except in Kermesidae and some Coccidae) (Hodgson 2020). However, Beardsley (1969) was the first to elevate *Puto* to family level based on the morphological characteristics of the adult male; this action was accepted by many coccoidologists (Miller and Miller 1993; Williams et al. 2011; Vea and Grimaldi 2016; Hodgson 2020; Choi and Lee 2022; Powell and Miller 2024), and it was considered to be a member outside of the traditional “neococcoid” lineage based on the molecular markers or morphology of the male adult (Gullan and Cook 2007; Williams et al. 2011; Vea and Grimaldi 2016; Hodgson 2020; Choi and Lee 2022). Phylogenetic trees reconstructed based on male morphological characteristics or combined molecular and morphological data revealed that Putoidae is more closely related to Phenacoleachiidae and Steingeliidae (Hodgson 2002; Hodgson and Foldi 2005; Vea and Grimaldi 2016). However, this relationship remains unresolved, as nucleotide sequence analyses recover Putoidae and Ortheziidae as a sister group (Gullan and Cook 2007).

The mitochondria are important functional organelles of eukaryotes (Boore et al. 2005). Mitochondrial genomes have been widely used for phylogenetic reconstruction and evolutionary analysis across various groups within insects, such as Aleyrodidae (Song et al. 2022), Hymenoptera (Wang et al. 2021; Xing et al. 2022), Mantodea (Lin et al. 2022), Psocodea (Saenz Manchola et al. 2021) and Trichoptera (Ge et al. 2022). Similarly, they are also used to reconstruct the phylogenetic relationship of neococcoids in Coccomorpha (Xu et al. 2023). However, to date, the mitochondrial genomes of scale insects recorded in GenBank have been extremely limited, with only 23 species from eight families sequenced. Among these, only Matsucoccidae and Monophlebidae belong to the archaeococcoids, while the remaining six families (Pseudococcidae, Eriococcidae, Kerriidae, Cerococcidae, Aclerdidae and Coccidae) all belong to the neococcoids. The mitochondrial genomes of scale insects consistently exhibit high A+T content, demonstrate pronounced variability in gene arrangement patterns, and frequently contain tRNAs without the dihydrouridine (DHU) arm or the (TΨC) T arm (Deng et al. 2019; Hou et al. 2023; Lu et al. 2023; Xu et al. 2023).

However, whether the phylogenetic position of Putoidae inferred from mitochondrial genes is consistent with that based on nuclear genes or morphological characteristics remains to be further investigated. The present study sequenced and annotated the first complete mitogenome of the family Putoidae. The phylogenetic tree was then reconstructed based on the mitogenomic sequences of 18 other coccoid species. Furthermore, the study explored mitochondrial genome rearrangements within Coccomorpha, providing new insights into their phylogenetic relationships.

## ﻿Material and methods

### ﻿Sampling, genomic DNA extraction

The sample of *Putosinensis* for DNA extraction was collected on the trunk of *Linderacommunis* (D. Don) Merr. (Lauraceae) from Guizhou Province, China, and was preserved in 95% ethanol under -20 °C at the Beijing Forestry University, Beijing, P. R. China (BFUC). The total genomic DNA of two individuals was extracted with the TIANamp Genomic DNA Kit following the manufacturer’s instructions. The voucher specimens are deposited at Beijing Forestry University, Beijing, P. R. China (BFUC). The *COI* sequences were amplified using the primers C1-1554F and C1-2342R (Deng et al. 2012) and sequenced via the Sanger method on an ABI 3730xl sequencer to provide seed sequences for subsequent mitochondrial genome assembly.

The phylogenetic analysis included 30 species, comprising 18 species from Coccomorpha, nine species from three other infraorders of Sternorrhyncha, and three species of the order Thysanoptera. Except for the data on *Putosinensis*, all sequence data were obtained from the National Center for Biotechnology Information at https://www.ncbi.nlm.nih.gov (refer to Table 1).

**Table 1.** Mitogenomes of species used for phylogenetic analysis.

Order	Infraorder	Family	Species
Thysanoptera		Aeolothripidae	*Aeolothripsindicus* Bhatti
			*Franklinothripsvespiformis* (Crawford)
		Thripidae	*Thripshawaiiensis* (Morgan)
Hemiptera	Aleyrodomorpha	Aleyrodidae	*Aleyrodesshizuokensis* Kuwana
			*Bemisiatabaci* (Gennadius)
	Psyllomorpha	Carsidaridae	*Allocarsidarabakeri* Hollis
			*Paracarsidaragigantea* (Crawford)
		Psyllidae	*Cyamophilawillieti* (Wu)
		Aphalaridae	*Rhinocolaaceris* (Linnaeus)
	Aphidomorpha	Aphididae	*Aphisglycines* Matsumura
			*Myzuspersicae* (Sulzer)
			*Acyrthosiphonpisum* Harris
	Coccomorpha	Matsucoccidae	*Matsucoccusmatsumurae* (Kuwana)
		Monophlebidae	*Iceryapurchasi* Maskell
			*Coronaproctuscastanopsis* Li, Xu & Wu
		Putoidae	*Putosinensis* Zheng & Wu
		Pseudococcidae	*Paracoccusmarginatus* Williams & Granara de Willink
			*Phenacoccusaceris* (Signoret)
			*Phenacoccusmanihoti* Matile-Ferrero
		Eriococcidae	*Acanthococcuscoriaceus* (Maskell)
			*Apiomorphamunita* (Schrader)
		Kerriidae	*Albotachardinasinensis* Zhang
		Cerococcidae	*Antecerococcustheydoni* (Hall)
		Aclerdidae	*Nipponaclerdabiwakoensis* (Kuwana)
			*Aclerdatakahashii* Kuwana
		Coccidae	*Didesmococcuskoreanus* Borchsenius
			*Saissetiacoffeae* (Walker)
			*Parasaissetianigra* (Nietner)
			*Ceroplastesjaponicus* Green
			*Ceroplastesfloridensis* Comstock

### ﻿Mitogenome assembly and annotation

We sequenced the genome of *Putosinensis* using a next-generation sequencing method with Illumina Hiseq 2500 at Berry genomics (Beijing, China) with 6× sequencing depth and with 150 bp paired-end sequencing reads. An Illumina TruSeq library was constructed from total genomic DNA of a single species with an average insert size of 150 bp. After removing adapters and low-quality reads using fastp v. 0.20.0 (Chen et al. 2018), high-quality reads were used for reference-guided assembly with NOVOPlasty (Dierckxsens et al. 2017), using the mitochondrial genome of *Matsucoccusmatsumurae* as the reference and the *COI* sequence of *Putosinensis* as the bait.

There is no mitogenome available in GenBank for either *Phenacoccusaceris* or *Coronaproctuscastanopsis*. So, SRA data of *P.aceris* and *C.castanopsis* were downloaded from NCBI to obtain their mitogenomes. The IDBA-UD (Peng et al. 2012) was also used for assembly due to its ability to handle complex sequencing reads more effectively than NOVOPlasty.

### ﻿Mitogenome annotation and analysis

The protein-coding genes (PCGs) and tRNA genes were annotated using the MITOS2 (Donath et al. 2019) on the Galaxy platform (The Galaxy Community 2024), and their accurate positions were confirmed by comparing them with existing mitochondrial genomes of scale insects in MEGA v. 7.0.26 (Kumar et al. 2016). Nine tRNA genes could not be found by MITOS2. We identified nine missing tRNA genes (*trnA*, *trnN*, *trnC*, *trnQ*, *trnP*, *trnY*, *trnS1*, *trnS2*, *trnL1*) using ARWEN v. 1.2 (Laslett and Canback 2008) and the manual method, referencing the tRNA of other scale insects. The *rrnS* was detected by MITOS2, and the *rrnL* was determined by the upstream and downstream of tRNAs and alignment with the other scale insects. Base composition, PCG codon usage, and relative synonymous codon usage (RSCU) values were calculated using MEGA v. 7.0.26, and figures were generated using ggplot2 (Wickham 2011) in R v. 4.3.2 (R Core Team 2023). The effective number of codons (ENC), the codon bias index (CBI), GC content, and the GC content of the third codon positions were calculated by codonW, and the number of synonymous substitutions per synonymous site (Ks), the number of nonsynonymous substitutions per nonsynonymous site (Ka) were calculated using the FMutSel model in PAML (Yang 2007), correcting for high AT content-induced nonsynonymous mutations, with *Aphisglycines* as the reference. Base composition skew values were computed as follows: AT skew = (A-T)/(A+T) and GC skew = (G-C)/(G+C) (Perna and Kocher 1995).

### ﻿Phylogenetic analysis

The entire workflow of the phylogenetic analysis was conducted using PhyloSuite v. 1.2.2 (Zhang et al. 2020). A dataset was compiled by merging 13 protein-coding genes within PhyloSuite. Phylogenetic relationship was inferred using maximum likelihood (ML) and Bayesian inference (BI). The sequences of protein-coding genes were aligned using the codon alignment model and G-INS-I (accurate) strategy in MAFFT (Katoh and Standley 2013). The aligned sequences were further optimized using MACSE v. 2.03 (Ranwez et al. 2018) to handle frameshift mutations and stop codons, ensuring accurate codon-level alignment. This was followed by the removal of poorly aligned positions of each gene sequence using GBlocks (Talavera and Castresana 2007). Finally, the 13 protein-coding genes were concatenated in PhyloSuite v. 1.2.2. The optimal partitioning scheme for maximum likelihood and Bayesian inference, with the best partitioning scheme and evolutionary models (Suppl. material 1) for 13 pre-defined partitions, was selected using PartitionFinder2 (Lanfear et al. 2017). Subsequently, maximum likelihood phylogenies were inferred using IQ-TREE (Nguyen et al. 2015) under the Edge-linked partition model for 5000 ultrafast bootstraps (Minh et al. 2013), and the Shimodaira–Hasegawa-like approximate likelihood-ratio test (Guindon et al. 2010). Additionally, Bayesian inference phylogenies were inferred using MrBayes v. 3.2.7a (Ronquist et al. 2012) under the partition model, with 2 parallel runs of 2,000,000 generations. The initial 25% of sampled data was discarded as burn-in.

## ﻿Results

### ﻿General features and nucleotide composition

**Figure 1.**
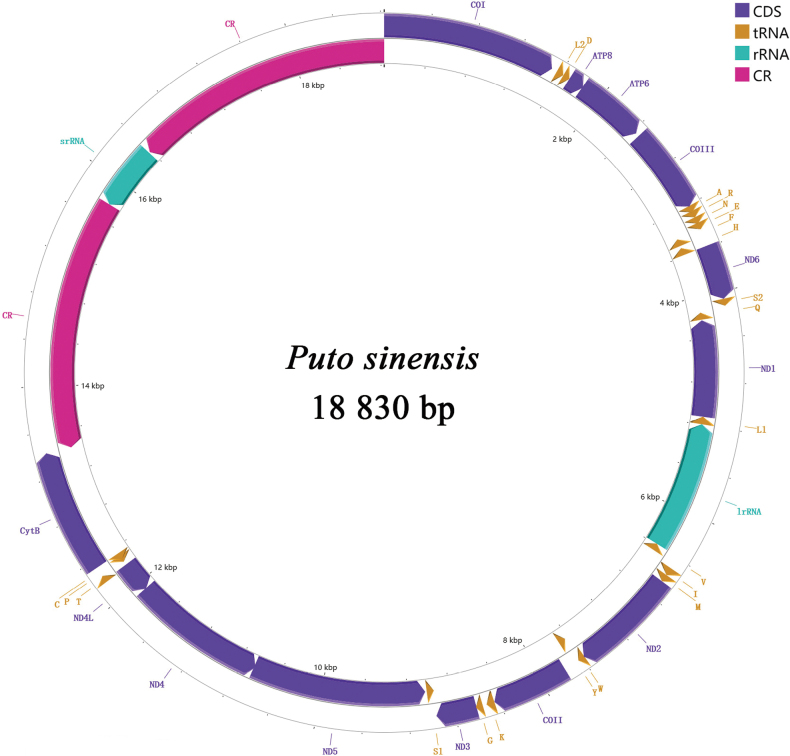
Circular maps of the mitogenomes of *Putosinensis*. The genes in the outer circle are located on the major (J) strand, while those in the inner circle are on the minor (N) strand. The tRNA genes are denoted by single-letter abbreviations corresponding to the amino acids they encode.

The mitochondrial genome of *Putosinensis* has 18,830 bp (Fig. 1), contains 37 typical genes, and has an A+T content of 90.7% (Table 2), consistent with a strong bias toward A+T. Fourteen genes are encoded by the minor (N) chain, including two rRNA (*srRNA*, *lrRNA*), four protein-coding genes (*ND1*, *ND4*, *ND4L*, *ND5*), and eight tRNAs (*trnF*, *trnH*, *trnQ*, *trnL1*, *trnV*, *trnY*, *trnP*, *trnC*). The remaining nine protein-coding genes (*ATP6*, *ATP8*, *COI*, *COII*, *COIII*, *cytb*, *ND2*, *ND3*, *ND6*) and 14 tRNA genes (*trnL2*, *trnD*, *trnA*, *trnR*, *trnN*, *trnE*, *trnS1*, *trnI*, *trnM*, *trnW*, *trnK*, *trnG*, *trnS2*, *trnT*) are situated on the major (J) chain. There are 15 overlapping regions, ranging from 1 to 22 bp, and 14 intergenic regions, ranging from 1 to 72 bp (Table 3).

**Table 2.** The nucleotide composition of the mitochondrial genome.

Feature	%T(U)	%C	%A	%G	%A+T	AT Skew	GC Skew
Whole genome	44.6	6.8	46.2	2.4	90.7	0.017	-0.475
Protein-coding genes	47.8	6.1	41.2	5.0	88.9	-0.074	-0.096
First codon position	37.9	5.6	50.2	6.3	88.1	0.141	0.057
Second codon position	56.5	7.4	29.9	6.2	86.4	-0.308	-0.083
Third codon position	48.9	5.2	43.4	2.5	92.3	-0.060	-0.353
Protein-coding genes J-strand	45.3	8.3	42.3	4.1	87.6	-0.035	-0.346
First codon position	33.0	7.7	54.3	5.0	87.3	0.244	-0.211
Second codon position	54.8	10.0	28.9	6.3	83.7	-0.309	-0.229
Third codon position	48.2	7.3	43.6	0.9	91.8	-0.051	-0.781
Protein-coding genes N-strand	51.6	2.4	39.4	6.5	91.1	-0.134	0.464
First codon position	45.6	2.3	43.7	8.4	89.4	-0.021	0.572
Second codon position	59.2	3.1	31.5	6.2	90.8	-0.306	0.333
Third codon position	50.1	1.8	43.0	5.1	93.0	-0.075	0.474
tRNA genes	44.6	3.1	47.9	4.4	92.5	0.036	0.172
tRNA genes J-strand	44.1	4.1	48.3	3.6	92.4	0.045	-0.065
tRNA genes N-strand	45.5	1.4	47.3	5.8	92.8	0.020	0.600
rRNA genes	46.3	2.4	44.6	6.8	90.8	-0.018	0.479

**Table 3.** The mitochondrial genome structure of *Putosinensis*.

Genes	Strand	Location	Length	Anticodon and its location	Start codon	Stop codon	Intergenic nucleotides
*COI*	J	1–1551	1551	-	ATT	TAA	0
*trnL2*	J	1560–1623	64	TAA 1592–1594	-	-	8
*trnD*	J	1626–1685	60	GTC 1663–1665	-	-	2
*ATP8*	J	1686–1826	141	-	ATT	TAA	0
*ATP6*	J	1820–2431	612	-	ATG	TAA	-7
*COIII*	J	2463–3215	753	-	ATG	TAA	31
*trnA*	J	3200–3275	76	TGC 3237–3239	-	-	-15
*trnR*	J	3268–3312	45	TCG 3294–3296	-	-	-4
*trnN*	J	3322–3378	57	GTT 3343–3345	-	-	9
*trnE*	J	3376–3431	56	TTC 3406–3408	-	-	-3
*trnF*	N	3432–3496	66	GAA 3449–3451	-	-	0
*trnH*	N	3518–3587	69	GTG 3552–3554	-	-	21
*ND6*	J	3586–4065	480	-	ATT	TAA	-2
*trnS1*	J	4068–4124	57	TGA 4085–4087	-	-	2
*trnQ*	N	4152–4205	54	TTG 4186–4188	-	-	27
*ND1*	N	4218–5120	903	-	ATT	TAA	12
*trnL1*	N	5120–5194	75	TAG 5155–5157	-	-	-1
*lrRNA*	N	5195–6388	1194	-	-	-	0
*trnV*	N	6389–6452	64	TAC 6416–6418	-	-	0
*trnI*	J	6504–6573	70	GAT 6539–6541	-	-	51
*trnM*	J	6574–6639	65	CAT 6607–6609	-	-	0
*ND2*	J	6640–7581	942	-	ATT	TAA	0
*trnW*	J	7580–7635	56	TCA 7612–7614	-	-	-2
*trnY*	N	7634–7704	71	GTA 7668–7670	-	-	-2
*COII*	J	7777–8448	672	-	ATA	TAA	72
*trnK*	J	8450–8519	70	TTT 8510–8512	-	-	1
*trnG*	J	8557–8612	56	TCC 8587–8589	-	-	37
*ND3*	J	8610–8963	354	-	ATA	TAA	-3
*trnS2*	J	8959–9012	58	TCT 8986–8988	-	-	-5
*ND5*	N	9034–10662	1629	-	ATT	TAA	17
*ND4*	N	10650–11918	1269	-	ATT	TAA	-13
*ND4L*	N	11914–12207	294	-	ATT	TAA	-5
*trnT*	J	12190–12248	59	TGT 12222–12224	-	-	-18
*trnP*	N	12247–12303	57	AGG 12269–12271	-	-	-2
*trnC*	N	12282–12321	40	GCA 12302–12304	-	-	-22
*cytb*	J	12336–13418	1083	-	ATG	TAA	14
*srRNA*	N	15769–16377	609	-	-	-	2350

### ﻿Protein-coding genes

**Figure 2.**
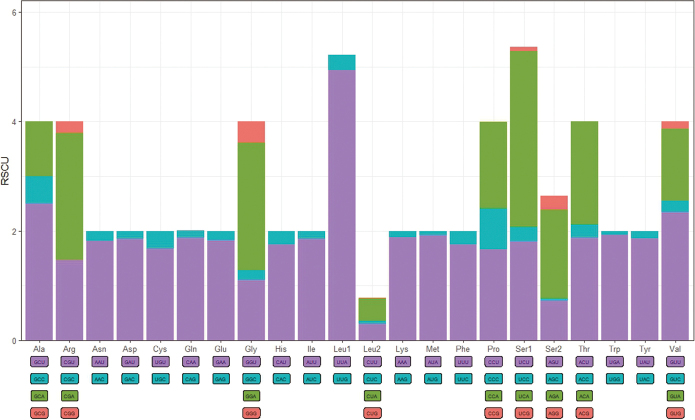
The relative synonymous codon usage (RSCU) in the mitogenomes of *Putosinensis*. Codon families are labeled on the x-axis.

The mitochondrial genome of *Putosinensis* comprises 13 protein-coding genes, with a length of 10,700 bp, and features a high A+T content of 88.9%. The relative synonymous codon usage (RSCU) is depicted in Fig. 2. In this mitogenome, codons ending with A or T were preferred for each amino acid. All start codons of protein-coding genes consist of ATN, and all terminate with the stop codon TAA. The usage of codons for protein-coding genes is presented in Table 4, with the most frequently used codons ranked in descending order as follows: ATT, ATA, TTA, AAT, and TTT.

**Table 4.** Codon usage in *Putosinensis*.

Amino acid	Codon	Count	RSCU	Amino acid	Codon	Count	RSCU
Phe	UUU(F)	325	1.75	Ser	UCU(S)	48	1.81
	UUC(F)	47	0.25		UCC(S)	7	0.26
Leu	UUA(L)	406	4.93		UCA(S)	85	3.21
	UUG(L)	24	0.29		UCG(S)	2	0.08
Leu(c)	CUU(L)	25	0.3	Ser(s)	AGU(S)	19	0.72
	CUC(L)	4	0.05		AGC(S)	1	0.04
	CUA(L)	34	0.41		AGA(S)	43	1.62
	CUG(L)	1	0.01		AGG(S)	7	0.26
Ile	AUU(I)	522	1.85	Thr	ACU(T)	24	1.88
	AUC(I)	41	0.15		ACC(T)	3	0.24
Met	AUA(M)	504	1.92		ACA(T)	24	1.88
	AUG(M)	21	0.08		ACG(T)	0	0
Val	GUU(V)	34	2.34	Ala	GCU(A)	10	2.5
	GUC(V)	3	0.21		GCC(A)	2	0.5
	GUA(V)	19	1.31		GCA(A)	4	1
	GUG(V)	2	0.14		GCG(A)	0	0
Tyr	UAU(Y)	238	1.86	Cys	UGU(C)	10	1.67
	UAC(Y)	18	0.14		UGC(C)	2	0.33
	UAA(*)	69	1.79	Trp	UGA(W)	57	1.93
	UAG(*)	8	0.21		UGG(W)	2	0.07
His	CAU(H)	28	1.75	Arg	CGU(R)	7	1.47
	CAC(H)	4	0.25		CGC(R)	0	0
Gln	CAA(Q)	30	1.88		CGA(R)	11	2.32
	CAG(Q)	2	0.13		CGG(R)	1	0.21
Asn	AAU(N)	375	1.82	Pro	CCU(P)	22	1.66
	AAC(N)	38	0.18		CCC(P)	10	0.75
Lys	AAA(K)	157	1.89		CCA(P)	21	1.58
	AAG(K)	9	0.11		CCG(P)	0	0
Asp	GAU(D)	37	1.85	Gly	GGU(G)	17	1.1
	GAC(D)	3	0.15		GGC(G)	3	0.19
Glu	GAA(E)	44	1.83		GGA(G)	36	2.32
	GAG(E)	4	0.17		GGG(G)	6	0.39

**Figure 3.**
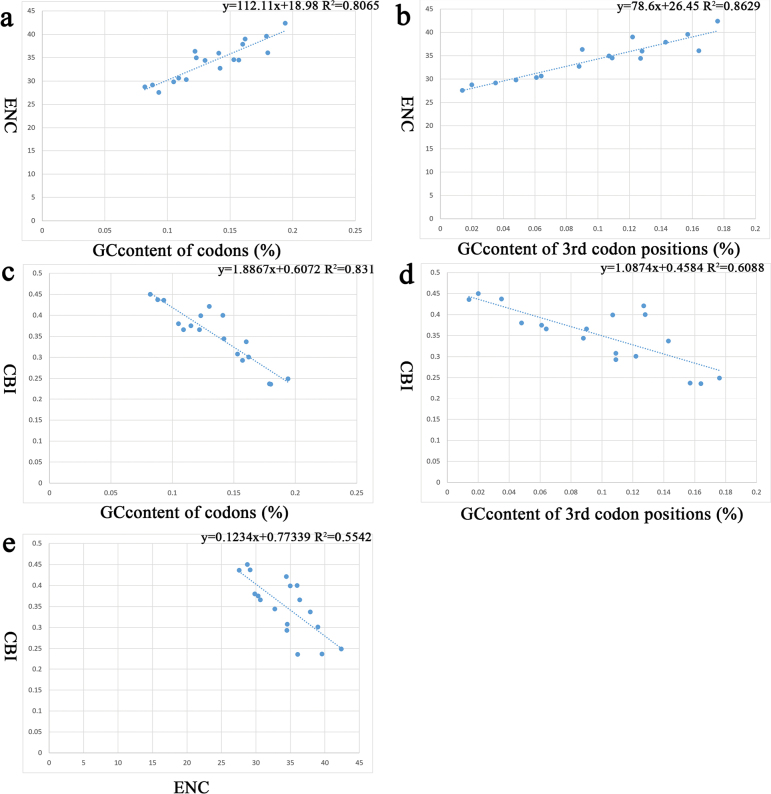
Evaluation of codon bias across 18 coccoid mitogenomes. **a.** Relevance of GC content of codons to ENC; **b.** Relevance of GC content of 3^rd^ codons positions to ENC; **c.** Relevance of GC content of codons to CBI; **d.** Relevance of GC content of 3^rd^ codon positions to CBI; **e.** Relevance of ENC to CBI.

We investigated codon usage bias through three metrics: CBI (codon bias index), ENC (effective number of codons) and GC content (including third codon positions, GC3) (Fig. 3a–e). The results revealed a positive correlation between ENC and the GC content of codons, as well as the GC content of the third codon positions (Fig. 3a, b). Conversely, CBI exhibits a negative correlation with both the GC content of codons and the GC content of the third codon positions (Fig. 3c, d). Similarly, a negative correlation was observed between ENC and CBI (Fig. 3e). This finding indicates that AT-rich genomes exhibit stronger codon usage bias.

**Figure 4.**
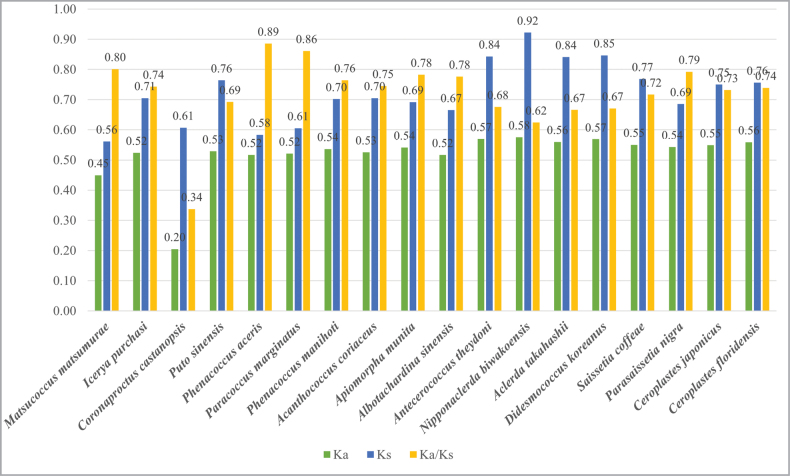
The Ka/Ks ratios for 13 protein-coding genes in the mitogenomes of 18 coccoid species compared with *Aphisglycines* as the reference.

By calculating the rates of nonsynonymous substitutions (Ka), synonymous substitutions (Ks), and the Ka/Ks ratio for the protein-coding genes of *Putosinensis* and 17 other species of scale insects (Fig. 4), it was found that the Ka/Ks ratios were less than one, indicating that the protein-coding genes of mitochondrial genomes are under purifying selection.

### ﻿tRNA genes

**Figure 5.**
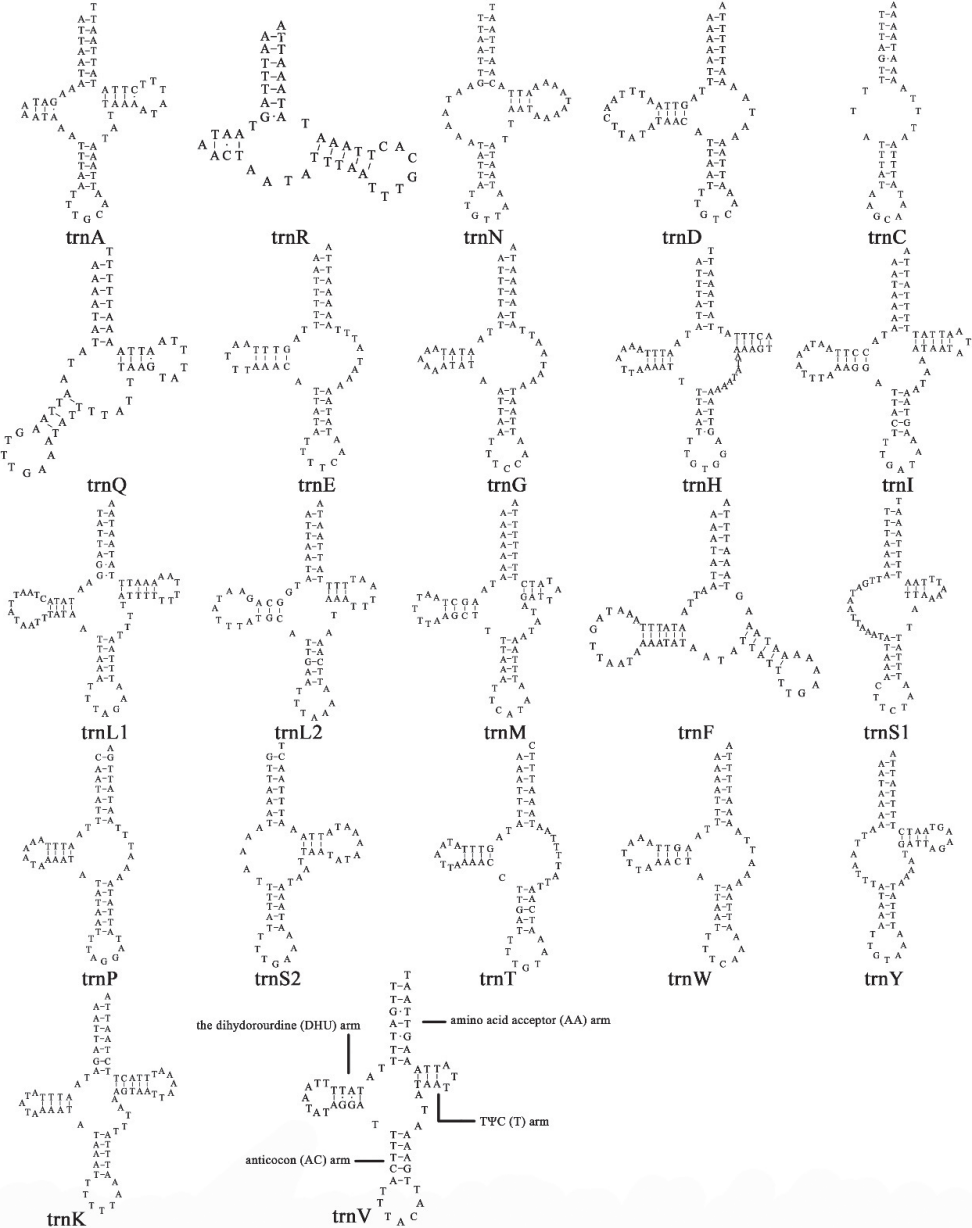
Secondary structures of the transfer RNA genes (tRNAs) in *Putosinensis* mitogenome inferred using MITOS2 and ARWEN v. 1.2. The tRNA genes are represented by the amino acid abbreviations.

Twenty-two tRNA genes were identified in *Putosinensis*, and the secondary structures are shown in Fig. 5. The total length of tRNAs was 1318 bp, with 45 bp to 75 bp in size and A+T content of 92.5%. Only a few tRNA genes exhibit a complete cloverleaf secondary structure (*trnA*, *trnH*, *trnI*, *trnL1*, *trnL2*, *trnM*, *trnK*, *trnV*), with eight lacking the T arm (*trnR*, *trnD*, *trnE*, *trnG*, *trnF*, *trnP*, *trnT*, *trnW*), five lacking the DHU arm (*trnN*, *trnQ*, *trnS1*, *trnS2*, *trnY*), and *trnC* lacking both the DHU and T arms.

Typical cloverleaf secondary structures can be formed by only a few tRNA genes, while the majority of tRNA genes are truncated.

The *rrnL* was located between *trnL1* and *trnV*, and the *rrnS* was located between two control regions. The length of *rrnL* with 1, 195 bp, and the length of *rrnS* with 609 bp. The *rrnL* had a high AT content, with 91%, and the *rrnS* also had a high AT content, with 90.5%.

### ﻿Gene rearrangements

**Figure 6.**
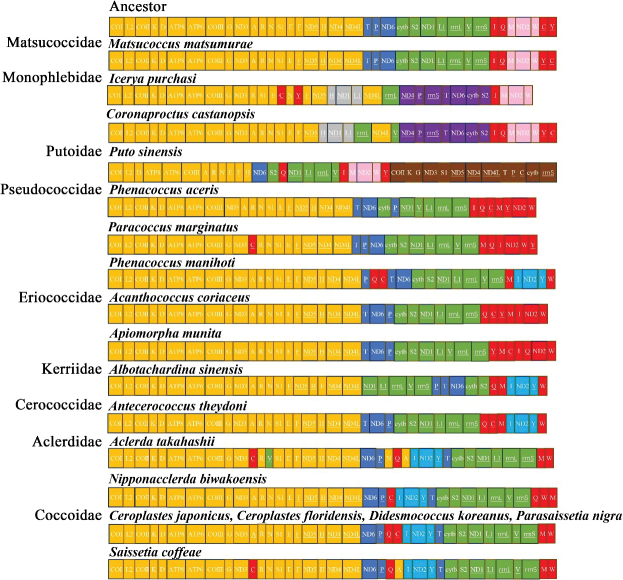
Gene orders in the ancestor and scale insect mitogenomes. The order was divided into four relatively conserved gene regions coloured with yellow, blue, green and (red+pink). The tRNA genes are denoted by single-letter abbreviations corresponding to the amino acids they encode.

The mitogenomes of all previously sequenced coccoid species have been rearranged (Fig. 6). Especially, *Putosinensis* has significant gene rearrangements of the mitogenome, compared to the putative ancestral arrangement. Five genes (*trnS1*, *ND5*, *trnC*, *cytb*, and *rrnS*) were rearranged with three gene clusters (*COII*-*trnK*, *trnG*-*ND3*, and *ND4*-*ND4L*-*trnT*-*trnP*), forming a new gene block, *COII*-*trnK*-*trnG*-*ND3*-*trnS1*-*ND5*-*ND4*-*ND4L*-*trnT*-*trnP*-*trnC*-*cytb*-*rrnS* (brown in Fig. 6). Besides, the gene arrangement of Matsucoccidae remains nearly identical to the ancestral one, with the only rearrangement being the relocation of *trnY* from downstream to upstream of *trnC*. In the family Monophlebidae, the *ND1*-*trnL1* gene cluster has been rearranged downstream of the *trnH*, forming a new gene cluster, *trnH*-*ND1*-*trnL1* (gray in Fig. 6). Additionally, the genes *ND4*, *trnP*, *rrnS*, *trnT*, *ND6*, *cytb*, and *trnS2* formed another novel cluster, *ND4*-*trnP*-*rrnS*-*trnT*-*cytb*-*trnS2* (purple in Fig. 6). The *trnM*-*ND2*-*trnW* (pink in Fig. 6) gene cluster was present in all four non-neococcoid species examined in this study. In contrast, the gene cluster *trnI*-*ND2*-*trnY* (light blue block in Fig. 6) was present in most neococcoid species.

### ﻿Phylogenetic analysis

**Figure 7.**
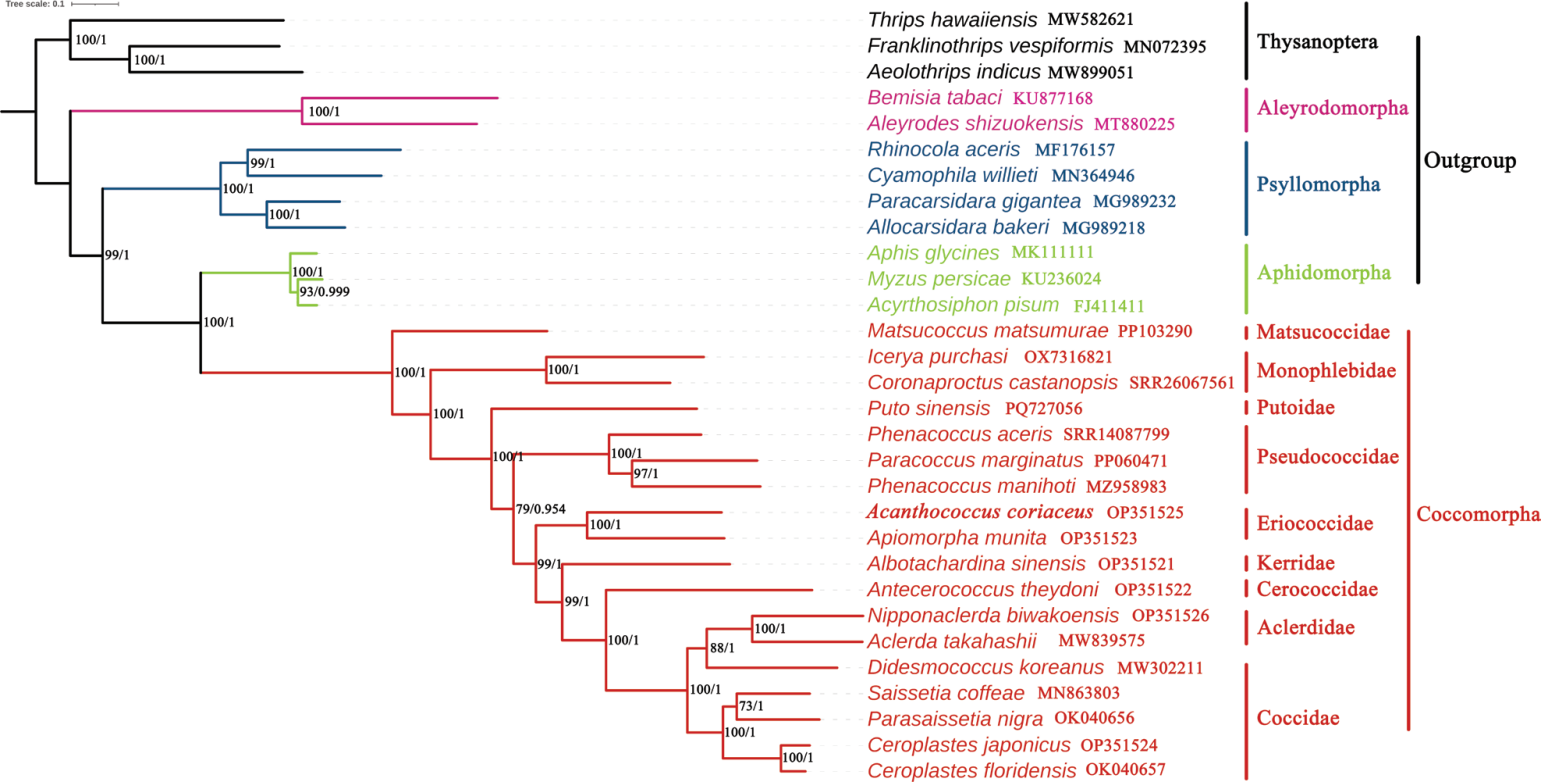
Phylogenetic trees of Coccomorpha were inferred using IQ-TREE and MrBayes from the 13 protein-coding genes. The values at nodes indicate maximum likelihood (ML) bootstrap values (left) and Bayesian posterior probabilities (right).

The phylogenetic analyses were conducted using sequences of 13 protein-coding genes from mitochondrial genomes of 27 species of Hemiptera and three species of Thysanoptera. Both Bayesian inference (BI) and maximum likelihood (ML) methods produced identical topologies (Fig. 7). Each suborder (Aleyrodomorpha, Psyllomorpha, Aphidomorpha and Coccomorpha) formed its own distinct clade with strong support. Among Coccomorpha, the archaeococcoids did not form a monophyletic group. Matsucoccidae and all other scale insects constituted a sister group, while Monophlebidae was sister to the clade (Putoidae + neococcoids), supported by 100% bootstrap values and 1.0 posterior probabilities. All neococcoids formed a well-supported clade (100% bootstrap values and 1.0 posterior probabilities) (Pseudococcidae + (Eriococcidae + (Kerriidae + (Cerococcidae + (Aclerdidae + Coccidae))))). Putoidae was sister to traditional neococcoids.

## ﻿Discussion

The conserved gene arrangement is recognized as a characteristic feature of mitochondrial genomes of insects (Boore 2000; Li et al. 2012; Cameron 2014). However, in some insect lineages, significant gene rearrangements occur, and these events can be used to infer phylogenetic relationships (Cameron 2014). The scale insects are a group characterized by extensive gene rearrangements. In this study, we identified an ancestral gene cluster, *trnM*-*ND2*-*trnW*, present in Matsucoccidae and Monophlebidae. Similarly, this ancestral gene arrangement is also found in Putoidae represented by *Putosinensis*, but absent in neococcoids. These genes were rearranged with other genes to form a novel gene order *trnI*-*ND2*-*trnY* in most neococcoid species, *trnY*-*ND2*-*trnW* in *Phenacoccusaceris* (Pseudococcidae), *trnI*-*ND2*-*trnW* in *Paracoccusmarginatus* (Pseudococcidae) and *Acanthococcuscoriaceus* (Eriococcidae), and *trnQ*-*ND2*-*trnW* in *Apiomorphamunita* (Eriococcidae). Although the mitogenomes of scale insects reported previously were very limited, these gene rearrangements (*trnI*-*ND2*-*trnY*, *trnY*-*ND2*-*trnW*, and *trnQ*-*ND2*-*trnW*) were regarded as apomorphies for neococcoids (Xu et al. 2023). And these rearrangements were not found in Putoidae. This indicates that Putoidae should be placed outside of the neococcoids.

Additionally, Putoidae was recovered as a sister group to neococcoids based on 13 protein-coding genes from the mitochondrial genome, further indicating that this family is more closely related to neococcoids than the two non-neococcoid families Matsucoccidae and Monophlebidae. The results from mitogenomic gene rearrangements and molecular phylogeny both support Putoidae as sister to neococcoids, consistent with evidence from molecular fragments, morphology of adult males, and multi-evidence analyses (Gullan and Cook 2007; Hodgson and Hardy 2013; Vea and Grimaldi 2016).

## ﻿Additional information

### Use of AI

No use of AI was reported.

### Author ORCIDs

Yu-ang Li https://orcid.org/0009-0004-8851-6796

Xinyi Zheng https://orcid.org/0000-0002-7396-7488

Han Xu https://orcid.org/0000-0001-7226-1742

San-an Wu https://orcid.org/0000-0002-9671-9401

### Data availability

All of the data that support the findings of this study are available in the main text or Supplementary Information.

## Supplementary materials

Supplementary material 1 Summary of partitioning scheme for Maximum Likelihood and Bayesian inference Data type docx https://binary.pensoft.net/file/1379238This dataset is made available under the Open Database License (http://opendatacommons.org/licenses/odbl/1.0/). The Open Database License (ODbL) is a license agreement intended to allow users to freely share, modify, and use this Dataset while maintaining this same freedom for others, provided that the original source and author(s) are credited.Yu-ang Li, Xinyi Zheng, Han Xu, San-an Wu
