# Supplementary material for: ﻿First mitogenome of the family Putoidae (Hemiptera, Coccomorpha) and its phylogenetic implications
Source: Zookeys. 2025 Jul 22;1247:1–18. doi: 10.3897/zookeys.1247.144896 (PMC12308203; doi:10.3897/zookeys.1247.144896)
Supplement: Supplementary material 1 — Summary of partitioning scheme for Maximum Likelihood and Bayesian inference [file zookeys-1247-001_article-144896__-s001.docx]

Table S1. Summary of partitioning scheme for Maximum Likelihood and Bayesian inference.

| Subset partitions | Sites | Best model for IQ-Tree | Best model for MrBayes |
| --- | --- | --- | --- |
| P1: (*ND3*, *ATP6*) | 933 | GTR+I+G | GTR+I+G |
| P2: (*ATP8*) | 135 | GTR+G | GTR+G |
| P3: (*COI*) | 1509 | GTR+I+G | GTR+I+G |
| P4: (*COII*) | 654 | GTR+I+G | GTR+I+G |
| P5: (*COIII*) | 753 | GTR+I+G | GTR+I+G |
| P6: (*cytb*) | 1080 | GTR+I+G | GTR+I+G |
| P7: (*ND1*) | 903 | GTR+I+G | GTR+I+G |
| P8: (*ND2*) | 903 | GTR+I+G | GTR+I+G |
| P9: (*ND4L*) | 264 | TVM+I+G | GTR+I+G |
| P10: (*ND4*, *ND5*) | 2796 | GTR+I+G | GTR+I+G |
| P11: (*ND6*) | 450 | GTR+I+G | GTR+I+G |
